# Supplementary material for: Across-species differences in pitch perception are consistent with differences in cochlear filtering
Source: eLife. 2019 Mar 15;8:e41626. doi: 10.7554/eLife.41626 (PMC6435318; doi:10.7554/eLife.41626)
Supplement: Figure 5—source data 1. [file elife-41626-fig5-data1.zip › Data/readme.rtf]

This folder contains the psychophysical data (PsychophysicalScores.mat) from Walker et al. (Elife submission 2018), as well as wav files of the stimuli presented to human (humanStimuli folder) and ferret (ferretStimuli folder) listeners on the task.


PsychophysicalScores.mat

This is a Matlab formatted data file generated using Matlab version 2015a. It contains the following variables.

ferretscores260 – a 3x6 matrix of the percent correct scores for 3 ferrets (rows) on trials in which one of 6 stimuli (columns) were presented. The '260' in the variable names indicates that the stimuli were presented with a reference of 260Hz.

ferretscores707 – a 4x6 matrix of the percent correct scores for 4 ferrets (rows) on trials in which one of 6 stimuli (columns) were presented. The '707' in the variable names indicates that the stimuli were presented with a reference of 707Hz.

humanscores - a 16x6 matrix of the percent correct scores for 16 human subjects (rows) on trials in which one of 6 stimuli (columns) were presented.

stimuli – a cell array of labels describing the 6 stimuli in the columns of ferretscores260, ferretscores707, and humanscores.


humanStimuli

This folder contains 10 Wav files, corresponding to the stimuli presented to humans for pitch classification in our psychophysical task.  The F0 and stimulus type are indicated in the file name.


ferretStimuli

This folder contains 24 Wav files, corresponding to the stimuli presented to ferrets for pitch classification in our psychophysical task.  The F0 and stimulus type are indicated in the file name.


Further questions about these files can be sent to Kerry Walker at kerry.walker@dpag.ox.ac.uk.


Kerry Walker, 2018
