# Supplementary material for: Across-species differences in pitch perception are consistent with differences in cochlear filtering
Source: eLife. 2019 Mar 15;8:e41626. doi: 10.7554/eLife.41626 (PMC6435318; doi:10.7554/eLife.41626)
Supplement: Supplementary file 1. [file elife-41626-supp1.docx]

Supplementary File 1a. Wilcoxon rank sum test comparing the power at F0 across all frequency channels in ferret and human cochlear simulations.

| Group | Median | Standard deviation | N (frequency channels) | Z value | p value | Effect size  $\frac{Z}{\sqrt{N_{1}+N_{2}}}$ |
| --- | --- | --- | --- | --- | --- | --- |
| Human | 0.163 | 0.343 | 1000 | 8.286 | 1.175 x 10^-16^ | 0.185 |
| Ferret | 0.346 | 0.355 | 1000 |  |  |  |

Supplementary File 1b. Paired t-test comparing ferrets’ performance in categorizing the F0 of the standard stimulus in silence and in the presence of a pink noise masker.

| Group | Mean | Standard deviation | N (ferrets) | t value | p value | Effect size  (Cohen’s d) |
| --- | --- | --- | --- | --- | --- | --- |
| Silence | 84.035 | 6.386 | 7 | 4.346 | 0.005 | 1.643 |
| Noise | 71.850 | 9.600 | 7 |  |  |  |

Supplementary File 1c. 1-sample t-test examining if ferrets’ performance in categorizing the F0 of the standard stimulus in the presence of a pink noise masker is above chance (50%).

| Mean | Standard deviation | N (ferrets) | t value | p value | Effect size  (Cohen’s d) |
| --- | --- | --- | --- | --- | --- |
| 71.850 | 9.600 | 7 | 6.025 | 0.001 | 2.277 |

Supplementary File 1d. Repeated measures (mixed) 3-way AVOVA examining ferrets’ performance on the categorization task, with stimulus type (standard and 4 probes), reference condition (260 and 707 Hz), and subject (3 ferrets per condition) as dependent variables.

| Dependent variable | Degrees of freedom | F value | p value | Effect size (Eta squared) |
| --- | --- | --- | --- | --- |
| Stimulus | 4 | 10.540 | 0.003 | 0.699 |
| Subject | 2 | 1.060 | 0.391 | 0.035 |
| Reference | 1 | 0.438 | 0.576 | 0.008 |

Supplementary File 1e. 2-way AVOVA examining ferrets’ performance on the 260 Hz reference categorization task, with stimulus type (standard and 4 probes) and subject (3 ferrets per condition) as dependent variables.

| Dependent variable | Degrees of freedom | F value | p value | Effect size (Eta squared) |
| --- | --- | --- | --- | --- |
| Stimulus | 4 | 9.794 | 0.004 | 0.817 |
| Subject | 2 | 0.366 | 0.704 | 0.015 |

Supplementary File 1f. 2-way AVOVA examining ferrets’ performance on the 707Hz reference categorization task, with stimulus type (standard and 4 probes) and subject (3 ferrets per condition) as dependent variables.

| Dependent variable | Degrees of freedom | F value | p value | Effect size (Eta squared) |
| --- | --- | --- | --- | --- |
| Stimulus | 4 | 14.194 | 0.0002 | 0.757 |
| Subject | 3 | 2.063 | 0.158 | 0.083 |

Supplementary File 1g. 2-way repeated measures AVOVA examining ferrets’ performance pooled across the 260 and 707Hz reference conditions, with stimulus type (standard and 4 probes) and subject (7 ferrets) as dependent variables.

| Dependent variable | Degrees of freedom | F value | p value | Effect size (Eta squared) |
| --- | --- | --- | --- | --- |
| Stimulus | 4 | 14.726 | 0.00003 | 0.664 |
| Subject | 6 | 0.962 | 0.471 | 0.065 |

Supplementary File 1h. Post-hoc pairwise comparisons between performance on the standard stimulus and each of the 4 probe stimuli. Performed on results of the above ANOVA, using Tukey’s HSD criterion.

| Probe type | Lower confidence interval | Mean | Upper confidence interval | p value |
| --- | --- | --- | --- | --- |
| Low harmonics | 4.706 | 13.989 | 23.272 | 0.001 |
| High harmonics | -9.448 | -0.165 | 9.118 | 1.000 |
| All harmonics in random phase | 4.112 | 13.395 | 22.678 | 0.002 |
| High harmonics in random phase | 9.339 | 18.622 | 27.905 | 0.0002 |

Supplementary File 1i. 2-way repeated measures AVOVA examining humans’ performance on the F0 categorization task, with stimulus type (standard and 4 probes) and subject (16 listeners) as dependent variables.

| Dependent variable | Degrees of freedom | F value | p value | Effect size (Eta squared) |
| --- | --- | --- | --- | --- |
| Stimulus | 4 | 36.999 | 1.443 x 10^-15^ | 0.521 |
| Subject | 15 | 5.050 | 2.812 x 10^-6^ | 0.267 |

Supplementary File 1j. Post-hoc pairwise comparisons between performance on the standard stimulus and each of the 4 probe stimuli. Performed on results of the above ANOVA, using Tukey’s HSD criterion.

| Probe type | Lower confidence interval | Mean | Upper confidence interval | p value |
| --- | --- | --- | --- | --- |
| Low harmonics | -1.026 | 8.824 | 18.673 | 0.101 |
| High harmonics | 17.412 | 27.261 | 37.110 | 1.029 x 10^-8^ |
| All harmonics in random phase | -7.432 | 2.417 | 12.266 | 0.959 |
| High harmonics in random phase | 23.818 | 33.667 | 43.516 | 9.922 x 10^-9^ |

Supplementary File 1k. 3-way repeated measures AVOVA examining performance on the F0 categorization task, with species (human and ferret), stimulus type (standard and 4 probes) and subject (16 listeners) as dependent variables. The interaction between species and stimulus type was also tested.

| Dependent variable | Degrees of freedom | F value | p value | Effect size (Eta squared) |
| --- | --- | --- | --- | --- |
| Stimulus | 4 | 36.963 | 0 | 0.303 |
| Species | 1 | 22.387 | 0.001 | 0.208 |
| Stimulus x species interaction | 4 | 14.802 | 3.412 x 10^-9^ | 0.121 |
| Subject | 21 | 4.534 | 3.200 x 10^-7^ | 0.196 |
