# Supplementary material for: Across-species differences in pitch perception are consistent with differences in cochlear filtering
Source: eLife. 2019 Mar 15;8:e41626. doi: 10.7554/eLife.41626 (PMC6435318; doi:10.7554/eLife.41626)
Supplement: Transparent reporting form [file elife-41626-transrepform.docx]

***eLife’s* transparent reporting form**

We encourage authors to provide detailed information *within their submission* to facilitate the interpretation and replication of experiments. Authors can upload supporting documentation to indicate the use of appropriate reporting guidelines for health-related research (see [EQUATOR Network](http://www.equator-network.org/%20)), life science research (see the [BioSharing Information Resource](https://biosharing.org/)), or the [ARRIVE guidelines](http://www.plosbiology.org/article/info:doi/10.1371/journal.pbio.1000412) for reporting work involving animal research. Where applicable, authors should refer to any relevant reporting standards documents in this form.

If you have any questions, please consult our Journal Policies and/or contact us: [editorial@elifesciences.org](mailto:editorial@elifesciences.org).

**Sample-size estimation**

- You should state whether an appropriate sample size was computed when the study was being designed
- You should state the statistical method of sample size computation and any required assumptions
- If no explicit power analysis was used, you should describe how you decided what sample (replicate) size (number) to use

Please outline where this information can be found within the submission (e.g., sections or figure legends), or explain why this information doesn’t apply to your submission:

An appropriate sample size was computed for our behavioural experiments. The power calculations are described in the Methods, on pages 23 (lines 448-450, and 463-464).

**Replicates**

- You should report how often each experiment was performed
- You should include a definition of biological versus technical replication
- The data obtained should be provided and sufficient information should be provided to indicate the number of independent biological and/or technical replicates
- If you encountered any outliers, you should describe how these were handled
- Criteria for exclusion/inclusion of data should be clearly stated
- High-throughput sequence data should be uploaded before submission, with a private link for reviewers provided (these are available from both GEO and ArrayExpress)

Please outline where this information can be found within the submission (e.g., sections or figure legends), or explain why this information doesn’t apply to your submission:

For the behavioural datasets in this manuscript, a biological replicate is an individual listener, described as a human or ferret “subject” in the manuscript. The number of subjects included in each analysis and figure is clearly defined throughout. A technical replicate is a behavioural “trial” in which the same stimulus is tested. The number of trials per stimulus is indicated on page 28 for ferret subjects (lines 589-591; 40 trials/ferret/probe and 1000 trials/ferret/standard) and page 29 for human listeners (lines 615-617; 40 trials/ferret/probe and 680 trials/ferret/standard).

Our criteria used for data exclusion are stated explicitly in the Methods section. On page 30 (lines 621-626), we explain that error correction trials were excluded from our analysis, as well as data from any testing session in which the subject scored less than 60% correct on standard trials.

**Statistical reporting**

- Statistical analysis methods should be described and justified
- Raw data should be presented in figures whenever informative to do so (typically when N per group is less than 10)
- For each experiment, you should identify the statistical tests used, exact values of N, definitions of center, methods of multiple test correction, and dispersion and precision measures (e.g., mean, median, SD, SEM, confidence intervals; and, for the major substantive results, a measure of effect size (e.g., Pearson's r, Cohen's d)
- Report exact p-values wherever possible alongside the summary statistics and 95% confidence intervals. These should be reported for all key questions and not only when the p-value is less than 0.05.

Please outline where this information can be found within the submission (e.g., sections or figure legends), or explain why this information doesn’t apply to your submission:

All error bars throughout the figures in the paper show mean and standard errors of the mean, as indicated in the methods (page 30, line 625). The number of subjects in each comparison is clear from the descriptions in the methods and results. Individual data points are plotted in Figure 5 – figure supplement 1, for the groups in Figure 5A and 5B, in which N is less than 20.

An alpha of 5% is used throughout the statistical tests in the manuscript, as indicated in our Methods (page 30, line 623).

When reporting the results of our experiments, we clearly indicate the statistical test used, the method of multiple test correction where appropriate (e.g. Tukey’s HSD), and the exact test statistic (e.g. t value) and p-value. We have included further details of all statistical tests performed in our experiments, including effect size calculations, as supplementary tables. These are referenced in the methods section (page 30; line 626).

(For large datasets, or papers with a very large number of statistical tests, you may upload a single table file with tests, Ns, etc., with reference to sections in the manuscript.)

**Group allocation**

- Indicate how samples were allocated into experimental groups (in the case of clinical studies, please specify allocation to treatment method); if randomization was used, please also state if restricted randomization was applied
- Indicate if masking was used during group allocation, data collection and/or data analysis

Please outline where this information can be found within the submission (e.g., sections or figure legends), or explain why this information doesn’t apply to your submission:

Ferrets were allocated to the 260 Hz and 707 Hz groups based on their availability at the time of testing. This is stated in the methods (page 27, line 549).

**Additional data files (“source data”)**

- We encourage you to upload relevant additional data files, such as numerical data that are represented as a graph in a figure, or as a summary table
- Where provided, these should be in the most useful format, and they can be uploaded as “Source data” files linked to a main figure or table
- Include model definition files including the full list of parameters used
- Include code used for data analysis (e.g., R, MatLab)
- Avoid stating that data files are “available upon request”

Please indicate the figures or tables for which source data files have been provided:

Complete data files of the stimuli presented in our experiments and the raw psychophysical scores used in all statistical analyses will be uploaded to Dryad archive as a part of the submission process, as instructed by eLife. Code used to replicate our model simulations and analyse our data will also be provided on Github.
